# Transcription factor AP-1 in esophageal squamous cell carcinoma: Alterations in activity and expression during Human Papillomavirus infection

**DOI:** 10.1186/1471-2407-9-329

**Published:** 2009-09-16

**Authors:** Showket Hussain, Alok C Bharti, Irfana Salam, Mohammad Akbar Bhat, Mohammad Muzaffar Mir, Suresh Hedau, Mushtaq A Siddiqi, Seemi Farhat Basir, Bhudev C Das

**Affiliations:** 1 Division of Molecular Oncology, Institute of Cytology & Preventive Oncology (ICMR), I-7, Sector-39, Noida, India; 2 Department of Clinical Biochemistry, Sher-I-Kashmir Institute of Medical Sciences, Soura, Srinagar, Jammu and Kashmir, India; 3 Department of Cardiovascular and Thoracic Surgery, Sher-I-Kashmir Institute of Medical Sciences, Soura, Srinagar, Jammu and Kashmir, India; 4 Department of Immunology and Molecular Medicine Sher-I-Kashmir Institute of Medical Sciences, Soura, Srinagar, Jammu and Kashmir, India; 5 Department of Biosciences, Jamia Millia Islamai; New Delhi, India; 6 Dr. B.R. Ambedkar Research Centre for Biomedical Research (ACBR), University of Delhi (North Campus), Delhi, India

## Abstract

**Background:**

Esophageal squamous cell carcinoma (ESCC) is a leading cause of cancer-related deaths in Jammu and Kashmir (J&K) region of India. A substantial proportion of esophageal carcinoma is associated with infection of high-risk HPV type 16 and HPV18, the oncogenic expression of which is controlled by host cell transcription factor Activator Protein-1 (AP-1). We, therefore, have investigated the role of DNA binding and expression pattern of AP-1 in esophageal cancer with or without HPV infection.

**Methods:**

Seventy five histopathologically-confirmed esophageal cancer and an equal number of corresponding adjacent normal tissue biopsies from Kashmir were analyzed for HPV infection, DNA binding activity and expression of AP-1 family of proteins by PCR, gel shift assay and immunoblotting respectively.

**Results:**

A high DNA binding activity and elevated expression of AP-1 proteins were observed in esophageal cancer, which differed between HPV positive (19%) and HPV negative (81%) carcinomas. While JunB, c-Fos and Fra-1 were the major contributors to AP-1 binding activity in HPV negative cases, Fra-1 was completely absent in HPV16 positive cancers. Comparison of AP-1 family proteins demonstrated high expression of JunD and c-Fos in HPV positive tumors, but interestingly, Fra-1 expression was extremely low or nil in these tumor tissues.

**Conclusion:**

Differential AP-1 binding activity and expression of its specific proteins between HPV - positive and HPV - negative cases indicate that AP-1 may play an important role during HPV-induced esophageal carcinogenesis.

## Background

Esophageal squamous cell carcinoma (ESCC) is one of the most common cancers in the world with extremely poor prognosis due to late presentation and rapid progression. It is eighth among the most common cancers worldwide and fifth most common cancer in developing countries [1]. ESCC shows a great variation in geographic distributions and the incidence rates are remarkably higher in distinct areas such as China, Singapore, Iran, France, South Africa, Puerto Rico, Chile, Brazil and northern and eastern Himalayan region. The wide geographical variation in the incidence reflects strong influence of environmental factors [2]. This cancer is also a major health problem in India; particularly in Kashmir valley and is associated with characteristic food and drinking habits such as drinking of hot salted tea which contains carcinogenic compounds like nitrosamines [3]. Besides salted tea, tobacco smoking (Hukka; local name Jajjer) is also very common in this area and is a potential risk factor for increased incidence of esophageal cancer [2,4,5]. Though recent reports have documented alterations of some oncogenes and tumor suppressor genes, the exact molecular and genetic basis of esophageal carcinogenesis still remains poorly understood [6].

Several studies have demonstrated infection of human papillomaviruses (HPVs) in esophageal cancer world over [7]. Though prevalence of this virus varies between 10 - 70% from one geographical region to other, the infection of high risk HPV (HR-HPV) types mainly HPV type 16 and HPV18 is found to be the most common in almost all parts of the world [8]. Our earlier study has also demonstrated that a significant proportion of esophageal cancer cases from Kashmir region are infected with HR-HPV type 16 [9]. This and other studies [7-9] indicate an oncogenic role of HR-HPV types in esophageal carcinogenesis. Since the virus does not have its own transcriptional machinery, the expression of its two transforming oncogenes, E6 and E7, depends primarily on availability of host cell transcription factors, particularly the Activator Protein-1 (AP-1) [10,11]. It has been demonstrated that a point mutation in the AP-1 consensus sequence within the binding site of upstream regulatory region (URR) of HPV16/18 leads to complete abolition of E6 and E7 gene expression [12]. Recent study also indicates that HPV infection may result in reciprocal alteration in AP-1 activity and its composition that could affect downstream gene expression and signaling leading to tumorigenesis of the infected cells [13] and better prognosis [14]. However, currently there is no study that defines the role of AP-1 in HPV induced esophageal carcinogenesis.

Considering the important role of AP-1 in a variety of epithelial cancers in general and those infected with HPV in particular, the present study has been carried out to investigate the possible relationship between HPV infection and expression profile of AP-1 family proteins in association with other etiological factors in the development of ESCC in Kashmir valley.

## Methods

### Patients and Specimens

A total of 150 tissue specimens comprising of 75 tumor tissues and 75 corresponding adjacent normal tissues as a control of ESCC, from 25 cases with no dysphagia and 50 cases of either grade I, II, and III dysphagia as classified earlier [15], were collected for analysis in the present study [Table 1]. None of these patients received any pre-operative radiation or chemotherapy. All samples were surgically resected and were collected at the Department of Cardio Vascular and Thoracic Surgery of Sher-I-Kashmir Institute of Medical Sciences, Soura, Srinagar, Kashmir (India). Tissue samples were divided into two parts; one part was sent to histopathological diagnosis and other half was stored in -70°C for molecular investigations. Histopathological grades and clinical staging were evaluated according to standard criteria [16] by two pathologists independently with 17 cases graded as well-differentiated squamous cell carcinoma (WDSSC), 45 as moderately-differentiated squamous cell carcinoma (MDSCC) and 13 as poorly-differentiated squamous cell carcinoma (PDSCC) whereas 46 patients were scored as stage I & II and 29 as stage III & IV. Only histopathologically confirmed cases were included for molecular analysis. Written informed consent was obtained from all the subjects included in the study and was carried out in accordance with the principles of the Helsinki Declaration. The study was approved by the Ethics Committee of the Hospital and the institute.

**Table 1:** Clinico-pathological and demographic characteristics and HPV status of Esophageal Squamous Cell Carcinoma cases investigated from Kashmir region.

Characteristics	Subgroup	Patients n = 75 (%age)
**Gender**		

Male		43(57)

Female		32(43)

**Age**		

40-60 years	Mean age (57.5)	47(63)

>60 years		28(37)

**Smoking Status**		

Smoker		51(68)

Non-smoker		24(32)

**Dysphagia**		

	Grade I, II, III	50(67)

	No Dysphagia	25(33)

**Location of tumor**		

	Upper	10(13)

	Middle	60(80)

	Lower	5(7)

**Clinical Stage**		

	Stage I & II	46(61)

	Stage III & IV	29(39)

**Histological Grade**		

	WDSCC	17(23)

	MDSCC	45(60)

	PDSCC	13(17)

**Consumption of hot salted tea/day**		

With sodium bicarbonate	1-3 cups	20 (27)
	
	3-6 cups	38(51)
	
	> 6 cups	10(13)

Without sodium bicarbonate		7(9)

**HPV status**		

Tumor Tissues	HPV L1 Positive	14 (19%)
	
	HPV16	14 (100%)
	
	Other HPVs	Nil

Normal Adjacent Tissues	HPV L1 Positive	Nil

### DNA extraction and PCR detection of HPV

High molecular-weight genomic DNA was isolated from tumor and normal adjacent tissue specimens by standard proteinase K digestion and a phenol-chloroform extraction procedure routinely followed in our laboratory [17]. PCR for detection of HPV16 and HPV18 DNA were carried out as described previously [18], using type-specific primers [HPV16 (1), 5-AAG GCC AAC TAA ATG TCA C-3; HPV16 (2), 5-CTG CTT TTA TAC TAA CCG G-3; HPV18 (1), 5-ACC TTA ATG AAA AAC CAC GA-3; HPV18 (2), 5-CGT CGT TTA GAG TCG TTC CTG-3]. Initially, all DNA samples were tested for the presence of HPV by using a pair of consensus primers located within the conserved L1 open reading frame (ORF) of the HPV genome (MY 11, 5-GCM CAG GGW CAT AAY AAT GC-3; MY 09, 5-CGT CCM ARR GGA WAC TGA TC-3; where M = A/C, W = A/T, Y = C/T, R = A/G). PCR was performed in a 25 μl reaction mix containing 100 ng DNA, 10 mM Tris-HCl (pH 8.4), 50 mM KCl, 1.5 mM MgCl_2_, 125 μM of each dNTP (dATP, dCTP, dGTP and dTTP), 5 pmol of each oligonucleotide primer and 0.5 U Taq DNA polymerase (Perkin-Elmer Biosystems, Foster City, CA, USA). The temperature profile used for amplification constituted an initial denaturation at 95°C for 5 min followed by 35 cycles with denaturation at 95°C for 30 sec, annealing at 55°C for 30 sec and extension at 72°C for 1 min, which was extended for 4 min in the final cycle. The oligonucleotide primers were synthesized in an automated Applied Biosystems DNA synthesizer (Model 381A; Applied Bio-systems, Foster City, CA, USA) using the phosphoramidite method and purified in high performance liquid chromatography (HPLC).

### Preparation of protein extract

Protein extracts from all ESCC biopsies (cancer and normal adjacent control) were prepared by the method of Dignam [19] with minor modification described earlier [14] Briefly, frozen tissues were minced and resuspended in ice-cold buffer A [20 mM HEPES (pH 7.6), 20% (v/v) Glycerol, 10 mM NaCl, 1.5 mM MgCl2, 0.2 mM EDTA, 1 mM DTT, 1 mM PMSF, 2 μg/ml Leupeptin and 10 μg/ml Aprotinin]. The lysates were microfuged at 4,000 rpm for 10 min at 4°C after incubating them for 15 min on ice. The supernatant was transferred in a new tube and designated as cytoplasmic extracts. The pellet containing isolated nuclei was resuspended in the 2 times pellet amount of extraction buffer B [20 mM HEPES (pH 7.6), 25% (v/v) Glycerol, 500 mM NaCl, 1.5 mM MgCl2, 0.2 mM EDTA, 1 mM DTT, 1 mM PMSF, 2 μg/ml Leupeptin and 10 μg/ml Aprotinin]. The extraction mixture was microfuged after 1 hr at 14,000 rpm at 4°C for 25 min. The resulting supernatant was designated as nuclear extract. The concentration of protein in the extracts was determined by standard Bradford method (Bio-Rad Laboratories, Inc.CA) and the extracts were stored at -70°C freezer until use.

### Electrophoretic mobility shift assay

For electrophoretic mobility shift assay (EMSA), the following oligonucleotides were used: AP-1 consensus sequence 5 - CGCTTGA*TGACTCA*GCCGGAA-3 (consensus binding sites are underlined and italicized), and Oct-1 consensus oligonucleotide 5-TGTCGA*ATGCAAAT*CACTAGAA-3. The oligonucleotide probes were synthesized in an Applied Biosystems DNA synthesizer using phosphoramidite chemistry. The above oligonucleotides were annealed and labelled with [γ^32^P] ATP (3,000 Ci/mmol; Jonaki, Hyderabad, India) by T4 polynucleotide kinase and gel purified in a 15% polyacrylamide gel [20]. The binding reaction was performed in a 25 μl reaction volume containing 50% glycerol, 60 mM HEPES (pH 7.9), 20 mM Tris-HCl (pH 7.9), 300 mM KCl, 5 mM EDTA, 5 mM DTT, 100 μg of BSA per milliliter, 2.5 μg of poly (dI-dC) and 10 μg of nuclear extract. After 5 min, 10,000 cpm of the [γ^32^P] ATP 5-end labelled double-stranded oligonucleotide probe was added and the incubation was continued for additional 25 min at room temperature. For monitoring AP-1 composition in supershift assays, 2 μg of polyclonal antibodies (Abs) directed against all the Jun and Fos family members (Santa Cruz Biotechnology Inc., Santa Cruz, CA) were added and the reaction mixture was further incubated for 1 hr at 4°C. The following Abs were used: anti-cJun (epitope corresponding to amino-terminal domain of mouse c-Jun p39); anti-JunB (epitope corresponding to carboxy terminal domain of mouse JunB); anti-JunD (epitope corresponding to carboxy terminus of mouse JunD); anti-cFos (epitope corresponding to a highly conserved domain of cFos p62 of human origin); anti-FosB (epitope corresponding to amino acids within the central domain of the FosB protein of mouse origin); anti-Fra-1 (epitope corresponding to amino terminus of Fra-1 of rat origin) and anti-Fra-2 (epitope corresponding to carboxy terminus of Fra-2 of human origin). The DNA-protein complexes were resolved on 4.5% non-denaturing polyacrylamide gel, PAGE (cross-linking ratio, 29:1), dried and exposed overnight to Phosphoimager (Fuji Film-Fla-5100) or Kodak X-Omat Films (Kodak India Ltd., India). The quantitative densitometric analysis was performed on shifted and super-shifted bands as a percent loss of band intensity indicating AP-1 binding in reaction having no antibody using Alpha Ease FC version 4.1.0 (Alpha Innotech Corporation. IL)

### Western blotting

Protein extracts (50 μg of protein per lane) were resolved in 10% polyacrylamide gel, electrotransferred to Immobilon-P membranes (Millipore Corporation, Bedford, MA), and probed with polyclonal rabbit antibodies of the corresponding family members of AP-1 as indicated in previous section. The incubation was carried out overnight in PBS supplemented with 5% skim milk powder, 0.05% Tween 20 (Sigma-Aldrich, CHEMIE GmbH, Germany), and different dilutions of respective antibodies. The bands were visualized with anti-rabbit immunoglobulin G (IgG) antibody conjugated with horseradish peroxidase using the Amersham ECL™ western blotting detection reagent kit (GE Healthcare). The blots were stripped and re-probed for β-actin levels to confirm equal loading and normalization. The expression levels of different AP-1 members were evaluated by densitometry using Alpha Digidoc version 4.1.0 (Alpha Innotech Corporation, IL) on a scale of 0-255 and the averaged pixel values were re-grouped for analysis on an arbitrary scale as strong => 50%; medium = 10-50%; weak = 1-10% and nil/not detectable < 1% as described earlier [21].

### Statistical Analysis

Statistical analysis on the data was performed using Epi-Info version 6.0 software (Center of Disease Control and Prevention, Atlanta, USA) and SigmaPlot version 10.0 (Systat Software, Point Richmond, CA). The association between HPV infection and expression profile with disease severity and clinico-pathological parameters was determined using the Fischer's Exact Test or Chi Square and Students t-test in order to compare the fold change of AP-1 binding between HPV positive and HPV negative ESCC cases. These tests were considered statistically significant when p ≤ 0.05.

## Results

A total of consecutive 75 surgically resected and histopathologically confirmed esophageal tumors along with corresponding normal adjacent tissues were analyzed to study the activity and expression of AP-1 in relation to HPV infection. Various clinico-epidemiological characteristics of esophageal squamous cell carcinoma cases from Kashmir valley and the status of their HPV infection is presented in Table 1. The cases investigated had slightly higher male representation (male to female ratio 1:0.74) with an average age of 57.5 years. With respect to tobacco consumption, 68% of the patients had smoking history and 67% of cases demonstrated various grades of dysphagia. Clinically, 39% cases had late presentation in either stage III or stage IV. Histopathologically, 60% of cases belonged to moderately differentiated squamous cell carcinoma Table 2. Patient stratification with respect to consumption of hot-salted tea containing sodium bicarbonate which is considered as a potent carcinogen in local Kashmiri population revealed consumption of three or more cups of salted tea per day by more than two third of the patient group.

**Table 2:** Association of Human Papillomavirus infection with clinicopathological, and other characteristics of ESCC cases

Characteristics (n = 75)	%age	HPV+Ve (n = 14) (%)	p-value
**Sex**			0.22
	
Male (n = 43)	57	6(14)	
	
Female (n = 32)	43	8(25)	

**Age**			0.89
	
40-60 years (n = 47)	63	9(19)	
	
>60 years (n = 28)	37	5(18)	

**Smoking Status**			0.03
	
Smoker (n = 51)	68	13(25)	
	
Non-smoker (n = 24)	32	1(4)	

**Dysphagia**			0.03
	
Grade I, II & III (n = 50)	67	13(26)	
	
No Dysphagia (n = 25)	33	1(4)	

**Location of tumor**			0.37
	
Upper (n = 10)	13	1(10)	
	
Middle (n = 60)	80	13(22)	
	
Lower (5)	7	-	

**Clinical Stage**			0.03
	
Stage I & II (n = 46)	61	5(11)	
	
Stage III & IV (n = 29)	39	9(31)	

**Histological Grade**			0.020
	
WDSCC (n = 17)	23	2(12)	
	
MDSCC (n = 45)	60	6(13)	
	
PDSCC (n = 13)	17	6(46)	

### Prevalence of Human Papillomavirus infection in ESCC

**Figure 1:**
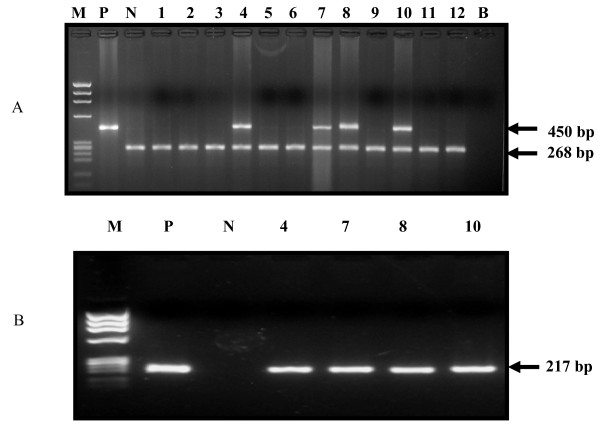
**(A&B): Detection of HPV infection by PCR in ESCC cases from Kashmir region**. Panel A, Representative ethidium bromide-stained 2% agarose gel showing presence of HPV infection in esophageal cancer as indicated by appearance of L1 consensus primer-generated amplimer of 450 bp along with amplification of 268 bp fragment of β-globin gene that was used as PCR internal control. Panel B, shows type-specific amplification of HR-HPV type 16 (217 bp) in HPV L1 positive samples of ESCC. P = positive control (HPV16DNA), N = negative control (Placental DNA), B = Blank, Lanes 1 to 12 are DNA samples from esophageal tumor biopsies, M = φX174 HaeIII-digested molecular weight marker.

Since HPV is considered as one of the important risk factor in esophageal carcinogenesis, tissue samples were screened by PCR for HPV infection using HPV L1consensus primers (Figure 1A), which revealed presence of HPV infection in 14 out of 75 tumor biopsies (19%) whereas, no HPV could be detected in any of the normal adjacent tissues. Subsequent PCR- based HPV typing using type-specific primers revealed that all 14 HPV L1 positive esophageal tumors were infected with HPV type 16 (Figure 1B). None of the HPV-infected esophageal tumors showed co-infection with other high- risk/low-risk HPV types.

To determine cofactors that may increase the risk of HPV infection in esophageal cancer, association of HPV infection with various clinico-pathological and demographic characteristics was examined [Table 2]. Majority of HPV infection was confined to patients with smoking history (p = 0.03) or having any grade (I, II or III) of dysphagia. A higher occurrence of HPV infection was also detected in advanced stage of cancer, both clinically (stage III and stage IV; p < 0.03) as well as histologically (WDSCC and PDSCC; p < 0.02). Most interestingly, more than 50% of HPV infection was confined to patients who were in poorly-differentiated state of the disease. Infection of HPV did not show any association with sex, age, or location of the tumor.

### Constitutive activation and DNA binding activity of AP-1 in ESCC in the absence or presence of HPV infection

**Figure 2:**
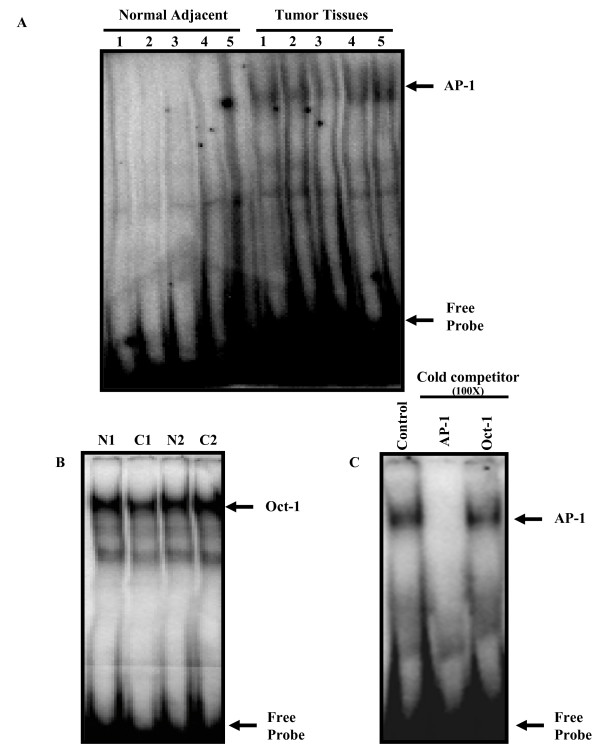
**(A-C): Bandshift assays showing constitutive activation and DNA binding specificity of AP-1 in esophageal cancer**. (A) nuclear proteins (10 μg) extracted from esophageal tumors (1-5) and their adjacent normal tissues were incubated with γ^32^P-labeled double stranded AP-1 binding probe and run on 4.5% non-denaturing PAGE. (B) uniform binding of Oct-1 in normal adjacent tissues and cancer. (C), binding specificity of AP-1 in esophageal cancer biopsies was evidenced by adding 100× molar excess of unlabeled homologous competitor, AP-1 oligo in competition with heterologous probe Oct-1 as indicated in methods. Arrows indicate the position of specific retarded bands.

**Figure 3:**
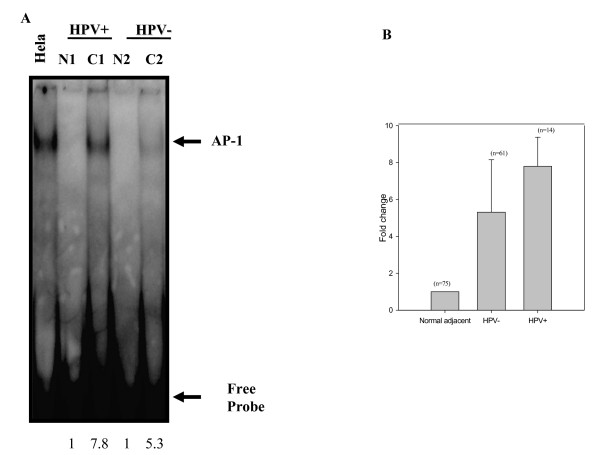
**(A&B): Gel shift analysis showing levels of active AP-1 DNA binding complexes present in HPV-positive and HPV-negative esophageal cancer biopsies**. (A) γ^32^P-labelled double stranded AP-1 oligonucleotides were co-incubated with 10 μg of nuclear protein extracts from HPV16 positive (C1), HPV negative (C2) esophageal tumor biopsies and corresponding normal adjacent tissues (N1, N2), analyzed on a 4.5% PAGE. The intensities of the bands were quantified as indicated in methods. Nuclear extracts of HeLa cells, which constitutively express active AP-1, was used as positive control. (B) Mean fold change in AP-1 binding activity in HPV negative and HPV positive esophageal cancer with respect to normal adjacent control tissues. Error bars indicate standard deviation, p-value 0.02 as compared to HPV negative tumor tissue. Arrows indicate the position of specific retarded bands.

To investigate the role of AP-1 in epithelial carcinogenesis, we analyzed the status of AP-1 DNA binding activity by gel shift assay in esophageal tumors and compared it with that of normal adjacent tissues. As shown in Figure 2A, the presence of constitutively active AP-1 DNA binding activity was detected in most of the esophageal tumors whereas, it was undetectable or very low in all corresponding normal adjacent tissues. Densitometric analysis of the shifted AP-1 band revealed variability in the degree of binding/activation in different cases ranging from 3 to 8 fold increase with respect to normal tissues. The specificity of AP-1 binding activity was confirmed by re-testing the samples for Oct-1 which is ubiquitously active in all cells and was used as an internal control by performing cold competition assay with 100-fold molar excess of homologous AP-1 and a heterologous Oct-1 cold probe (Figure 2B and 2C). In order to examine if HPV infection has any effect on AP-1 binding activity in esophageal cancer, as depicted in representative photograph in Figure 3A, we observed a differential activation of AP-1 in ESCC; an elevated AP-1 DNA-binding activity was significantly higher in HPV-positive tumors as compared to that in HPV-negative tumor tissues. These observations were reconfirmed by densitometric evaluation of gel shifted bands that showed significantly higher (p < 0.05) AP-1 binding activity in HPV-positive tumors than that of HPV-negative tumors (Figure 3B).

### Composition of functional AP-1 complex in HPV-negative and HPV-positive esophageal squamous cell carcinoma

**Figure 4:**
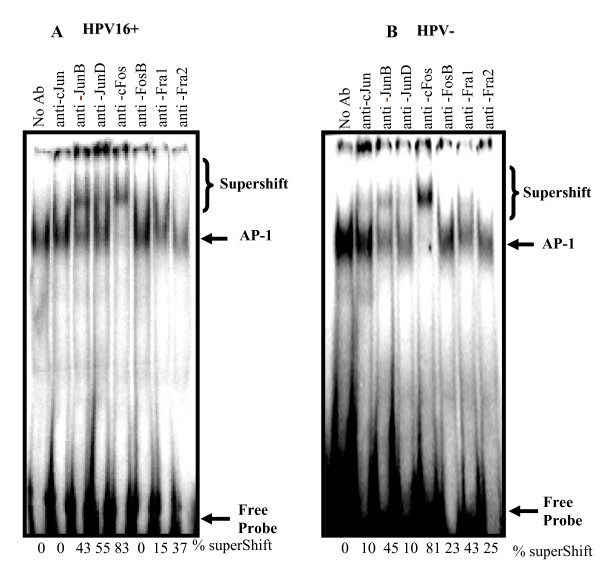
**(A&B): Band supershift assay showing composition of AP-1 complexes in HPV positive and HPV negative esophageal cancer**. 10 μg of nuclear protein extracts from HPV16-positive (A) or HPV-negative (B) ESCC biopsies were co-incubated with γ^32^P-labeled double stranded AP-1 probe. Oligonucleotides and specific antibodies (2 μg each) to all AP-1 family proteins as indicated and analyzed on a 4.5% PAGE. The intensities of the shifted and super-shited bands were quantified. Supershift was calculated as a percent loss of band intensity indicating AP-1 binding in reaction having no antibody.

Since functional AP-1 complex is constituted either by homo or hetrodimerization between different members of Jun and Fos family of proteins, we analyzed the composition of AP-1 complex in esophageal tumor tissues both in the presence and absence of HPV infection in a gel supershift assays by adding specific antibodies to all 7 members of AP-1 e.g. cJun, JunB, JunD, cFos, FosB, Fra-1 and Fra-2 (Figure 4A &4B). The supershift analysis revealed a preferential hetrodimerization between cFos and JunB instead of its canonical dimerization partner cJun which did not participate in AP-1 complex formation although it showed up-regulated expression in western blotting both in HPV positive as well as HPV negative tumors. In majority of HPV negative tumors more than 85% (53/61) of the supershifts had a JunB participation. Among the Fos family of proteins; mainly cFos was found to be the major contributor but Fra-1 also showed a minor participation as revealed in band supershift assays. No other Jun or Fos family members showed up in supershift assay (Figure 4B). On the other hand, HPV16-infected (n = 14) tumors also showed major participation of JunB and cFos but the most interesting observation was an exclusive participation of JunD in more than 70% of cases (10/14). In contrast, Fra-1 was completely absent in functional AP-1 activity (Figure 4A). In both HPV-positive and HPV-negative tumors cFos was the major partner contributing more than 90% participation in the DNA binding activity.

### Expression of AP-1 family proteins in esophageal squamous cell carcinoma in presence or absence of HPV infection

**Figure 5:**
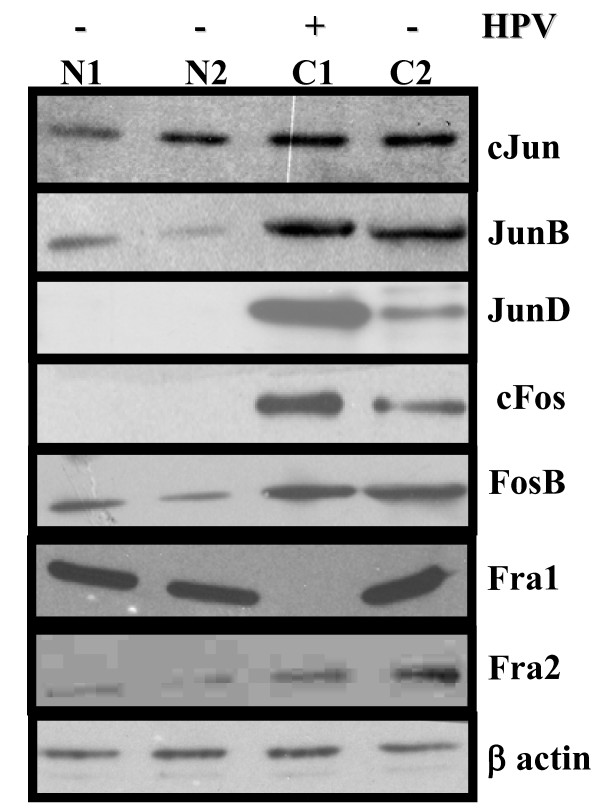
**Western blots showing expression pattern of AP-1 family of proteins in HPV positive and HPV negative esophageal cancer**. Protein extracts from HPV positive (C1) and HPV negative (C2) tumor biopsies as well as their corresponding adjacent tissue (N1, N2) were separated in 10% SDS-PAGE and detected by all specific antibodies of AP-1 family proteins as indicated. All the blots were stripped and reprobed for β-actin levels to confirm equal loading and the quantitation of bands was performed densitometrically as indicated in methods.

Western blotting experiments were performed to analyze the pattern and level of expression of all AP-1 family proteins (cJun, JunB, JunD, cFos, FosB, Fra-1 & Fra-2) in ESCC cases. Most of the AP-1 family members showed differential expression pattern in esophageal carcinoma tissues compared to their adjacent normal counterparts. While a very low or negligible expression of cJun, JunB, JunD, cFos, FosB, Fra-1 and Fra-2 was observed in normal adjacent tissues, the majority of them showed significantly elevated expressions in tumor tissues (Table 3 and Figure 5). In view of the impact of HPV infection on the binding activity and composition of functional AP-1 complex, we examined the expression pattern in HPV-positive and HPV-negative tumors. As shown in Figure 5 and Table 4, we observed a higher level of expression of JunD and cFos in HPV infected cases. Though the expression pattern of other members was higher, there was no significant change in expression pattern of these proteins with respect to HPV infection. Interestingly, unlike in other cancers, Fra-1 expression was diminished or completely lost in tumor tissues infected with HPV16 while HPV-negative tumors showed a very high expression of Fra-1 and also participates in DNA binding as revealed by supershift assay. The higher expression pattern of JunB, JunD, cFos are in concordance with gel shift assays that showed the participation of these proteins in AP-1 complex formation.

**Table 3:** Expression profile of AP-1 proteins in immunoblotting and densitometric analysis of their level of expression in normal adjacent controls and tumor tissues of ESCC.

AP-1 Family Member Protein	Normal (n = 75)				Cancer (n = 75)				p-value
	**Nil**	**Weak**	**Medium**	**Strong**	**Nil**	**Weak**	**Medium**	**Strong**	

**cJun**	10	23	42	-	-	5	24	46	0.001

**JunB**	49	16	8	2	2	9	30	34	0.001

**JunD**	53	20	2	-	-	7	21	47	0.001

**cFos**	69	6	-	-	-	-	13	62	0.001

**FosB**	4	8	36	27	2	14	29	30	0.52

**Fra-1**	35	12	19	9	11	17	10	37	0.003

**Fra-2**	5	5	28	37	11	16	18	30	0.002

Arbitrary level of expression in Immunoblotting: strong =>50%; medium = 10-50%; Weak = 1-10%; and Nil/undetectable =< 1%. Statistical calculation was done by grouping nil & weak, medium & strong together. Yates corrected chi-square/Fishers exact test were performed.

**Table 4:** AP-1 protein expression profile and their densitometric analysis in HPV negative and HPV positive cases of ESCC.

AP-1 Family Member Protein	HPV negative Cases (n = 61)				HPV positive Cases (n = 14)				p value
	**Nil**	**Weak**	**Medium**	**Strong**	**Nil**	**Weak**	**Medium**	**Strong**	

**cJun**	-	5	20	36	-	-	4	10	0.6

**JunB**	2	8	26	25	-	1	4	9	0.64

**JunD**	-	7	19	35	-	-	2	12	0.41

**cFos**	-	-	13	48	-	-	-	14	ND

**FosB**	2	13	23	23	-	1	6	7	0.2

**Fra-1**	-	14	10	37	11	3	-	-	0.001

**Fra-2**	8	10	15	28	3	6	3	2	0.03

Arbitrary level of expression in Immunoblotting: strong =>50%; medium = 10-50%; Weak = 1-10%; and Nil/undetectable =< 1%. Statistical calculation was done by grouping nil & weak, medium & strong together. Yates corrected chi-square/Fishers exact test were performed.

## Discussion

Although AP-1 has been demonstrated to play a crucial role in HPV - induced carcinogenesis and oncogenic HR-HPVs have been detected in a sizable number of ESCC, there is no study that describes the role of AP-1 in ESCCs. In the present study, we demonstrate for the first time, constitutive activation of AP-1 in ESCCs and change in binding partners that form active AP-1complex in ESCCs. ESCC with HPV infections constituted a significant proportion (19%; 14/75) and interestingly all these cases harbored the most prevalent high-risk HPV type 16. Constitutive activation of AP-1 leading to its high binding activity was observed in most of the esophageal tumors, irrespective of their clinical stage and histo - pathological grade; whereas normal adjacent tissues showed low or no AP-1 activation. Aberrant AP-1 activity is one of the most frequent mechanisms of tumor promotion in epithelial tissues irrespective of the tumor site and is mediated through activation of its upstream kinases such as ERK, JNK and p38 [22]. Similar to our observation, constitutively active AP-1 has also been observed in other epithelial cell malignancies [21,23-25]. But the mechanism(s) by which AP-1 or its upstream regulatory kinases get activated in ESCC is not known. Apart form physiological regulators like growth factors, cytokines or hormones, AP-1 activity is also induced by bacterial and viral infections as well as many carcinogens [26]. Recent study showed that HPV which is found in almost all cervical cancer, can also promote AP-1 activation to a significant level [13,21]. Apart from HPV, other infections such as *H. pylori*, prevalence of which is notably high in Kashmir region, are also known to induce AP-1 activation [27]. Another factor that could significantly contribute to constitutive activation of AP-1 is consumption of sodium-bicarbonate-brewed hot salted tea which is very widely consumed by majority of people in snow-capped Kashmir Valley [28]. This salted tea it is rich in carcinogenic N-nitroso compounds [3] that primarily act through AP-1 [11]. Interestingly, majority of ESCC cases investigated in the present study showed AP-1 activation had a high intake of salted tea.

Functional AP-1 complex is constituted either by homo- or hetero-dimerization between different members of Jun and Fos family of proteins [25]. Our results demonstrate a preferential participation of JunB and c-Fos in active AP-1 complex formation in almost all tumors irrespective of HPV infection. Interestingly, HPV positive tumors demonstrated an additional participation of JunD in AP-1 DNA binding complex. Also, Fra-1 which showed minor participation in HPV negative tumors was completely absent in HPV positive cases. Apart from formation of heterodimer between Jun and Fos proteins, homodimerization of Jun proteins is also known to form active AP-1 complex but complete absence of cJun in supershift assays indicate that cJun do not participate in functional AP-1 complex formation and hence no jun homodimer formation takes place in ESCC. It is interesting to note here that though c-Jun showed elevated expression in western blotting (see Figure 5), it does not participate in DNA binding activity. It is quite possible that even if a protein is overexpressed, it may not always participate in DNA binding activity and trans-activation possibly due to mutation within the binding sites or otherwise. It can also be speculated that in addition to posttranslational modifications of c-Jun, a simple competition with JunB [29] and JunD to bind with cFos, may also account for the exclusion of cJun from AP-1 complex in esophageal cancer. It is interesting to note that presence of active JunB/cFos dimers may provide a favorable niche for establishment of HPV infection and viral propagation as two AP-1 binding sites are present in URR of HPV18 that essentially require JunB containing hetrodimers for HPV transcription [12]. These observations, therefore, indicate presence of suitable co-operativity between the virus and the host for expression of viral oncogenes.

Most of the ESCC tissues irrespective of their clinical and histopathological grade showed a high expression of AP-1 family of proteins in tumor tissues as compared to their normal counterparts. Upregulated AP-1 activity is frequently associated with overexpression of its family members [30,31]. Since majority of AP-1 members are part of immediate early response genes and express differentially in non-neoplastic and neoplastic tissues as well as contribute both in early events of tumorigenesis and tumor progression [30,31], elevated AP-1 expression and activity appears to be generic carcinogenesis-associated event. Though it is well established that AP-1 regulates the expression of HPV oncogenes [10], recent observations indicate that HPV reciprocally modulate AP-1 expression and its activity [13]. We stratified expression levels of AP-1 which revealed a differentially higher expression of JunD and cFos proteins in HPV-positive tumors, whereas Fra-1 levels were characteristically low or nil in them. Fra-1 was, however, highly expressed in HPV- negative tumors. Over-expression of Fra-1 and cFos have been demonstrated in esophageal cancers in some sporadic studies [32,33]. On the contrary, an *in vitro *study demonstrated increase of cFos in HPV16-transformed cell lines is associated with decreased expression of its negative regulator Fra-1 as well as their reduced contribution in active DNA-binding activity [13]. In an ingenious experiment, overexpression of cFos in HPV 18 positive non-tumorigenic HeLa - Fibroblast hybrid 444 cells having low or no cFos but a very high amount of Fra-1 expression resulted in tumorigenic cells which showed complete loss of Fra-1 but a high expression of cFos [34]. These observations perfectly match with *in vivo *situations in HPV-infected esophageal tumors, where Fra-1 expression was diminished or completely absent. These observations, therefore, indicate a potential antagonistic effect of HPV infection on Fra-1. Fra-1 have been proposed to have tumor suppressor function as it is located in chromosome 11q13 region which is known to harbor tumor suppressor genes [35] and its over-expression have recently been shown to inhibit cell proliferation, induce apoptosis and reduce tumorigenesity [36]. Moreover, change in AP-1 composition resulting in elimination of Fra-1 has been found to be associated with enhanced tumorigenesity [13,34]. Thus, lack of Fra-1 appears to contributing to more aggressiveness of the disease, as majority of these patients were infected with HPV and in poorly-differentiated state. This is in sharp contrast to our recent observation in oral cancer, where presence of HPV but lack of Fra-1 expression was observed mainly in well-differentiated oral squamous cell carcinomas that showed better prognosis [37]. Thus, it appears that the role of Fra-1 as a tumor suppressor or oncogene may vary from tumor to tumor and on host-virus interactions.

Considering oncogenic role of HPVs [38] and their co-operative interaction with AP-1 signaling [10,13], infection with HPV has been implicated as a possible etiological factor in the development of squamous cell carcinoma of the esophagus. Our results demonstrated unlike cervical cancers where HR-HPV infection is an essential etiological factor, the percentage of cases with HPV infection in ESCC was small and accounted for about one fifth of total cases. Occurrence of HPV infection in esophageal cancer is variable and conflicting, ranging from complete absence to detection of up to 60 to 70% mainly of high - risk HPV types 16 and 18 [8,39]. Our earlier study on different subset of samples from the same region showed similar frequency of HPV16 infection [9]. Though considerable proportions of esophageal cancers have been shown to have HPV infection and that too of high risk types, the etiological role of HPV in these cancers is not established. It is suggested that HPV infection when present may act as a co-factor or have synergistic effects with environmental carcinogens in the genesis of ESCC and/or their progression. We also demonstrate a significant correlation of HPV infection with smoking habit and intake of nitrosated compounds in salted tea [Table 2]. The entry of HPV in esophageal cancers appears to be in later stages as the infection was more prevalent in clinically advanced tumors. Therefore, HPV may significantly influence the disease progression rather than initiation. In contrast to genital cancers, it has been shown that HPV infection may be associated with better prognosis [14] as it is found more in well-differentiated squamous cell carcinomas of the head and neck [40] and may positively influence the treatment outcome [41]. Therefore, determination of HPV status in esophageal cancer may also be helpful in predicting the treatment outcome.

## Conclusion

Present investigation provides evidence of a constitutively activated AP-1 in esophageal cancers and demonstrates involvement of JunB, JunD and cFos as major DNA binding partners whereas it clearly negates the role of canonical AP-1 partner, cJun. Expression of AP-1 proteins and their DNA-binding activity was found to differ between HPV positive and HPV negative tumors. Considering that HR-HPV infection causes better prognosis in head and neck cancers, present findings may provide clue(s) for better understanding of HPV-mediated esophageal carcinogenesis and development of treatment strategies.

## Competing interests

The authors declare that they have no competing interests.

## Authors' contributions

SH carried out all experiments and primary manuscript writing. ACB contributed in designing and interpretation of the study. IS collected samples and performed DNA extraction. MAB Gastroenterologist who collected data from patients and performed surgery. MMM senior scientist who contributed to critical revision of the manuscript. SH conceived and participated in the study. MAS conceived, design and acquisition of data. SFB senior scientist who oversaw the work and critical revision of the manuscript. BCD senior Professor, who oversaw and guaranteed the work and for conception, design and critical corrections of the manuscript. All authors read and approved the final manuscript.

## Pre-publication history

The pre-publication history for this paper can be accessed here:

http://www.biomedcentral.com/1471-2407/9/329/prepub
